# Residential area characteristics and disabilities among Dutch community-dwelling older adults

**DOI:** 10.1186/s12942-016-0070-8

**Published:** 2016-11-15

**Authors:** Astrid Etman, Carlijn B. M. Kamphuis, Frank H. Pierik, Alex Burdorf, Frank J. Van Lenthe

**Affiliations:** 1 Department of Public Health, Erasmus University Medical Centre, P.O. Box 2040, 3000 CA Rotterdam, The Netherlands; 2 Department of Human Geography and Spatial Planning, Utrecht University, Utrecht, The Netherlands; 3 Department of Urban Environment and Safety, TNO, Utrecht, The Netherlands

**Keywords:** Elderly, Mobility, Functioning, Limitations

## Abstract

**Background:**

Living longer independently may be facilitated by an attractive and safe residential area, which stimulates physical activity. We studied the association between area characteristics and disabilities and whether this association is mediated by transport-related physical activity (TPA).

**Methods:**

Longitudinal data of 271 Dutch community-dwelling adults aged 65 years and older participating in the Elderly And their Neighbourhood (ELANE) study in 2011–2013 were used. Associations between objectively measured aesthetics (range 0–22), functional features (range 0–14), safety (range 0–16), and destinations (range 0–15) within road network buffers surrounding participants’ residences, and self-reported disabilities in instrumental activities of daily living (range 0–8; measured twice over a 9 months period) were investigated by using longitudinal tobit regression analyses. Furthermore, it was investigated whether self-reported TPA mediated associations between area characteristics and disabilities.

**Results:**

A one unit increase in aesthetics within the 400 m buffer was associated with 0.86 less disabilities (95% CI −1.47 to −0.25; p < 0.05), but other area characteristics were not related to disabilities. An increase in area aesthetics was associated with more TPA, and more minutes of TPA were associated with less disabilities. TPA however, only partly mediated the associated between area aesthetics and disabilities.

**Conclusions:**

Improving aesthetic features in the close by area around older persons’ residences may help to prevent disability.

## Background

In ageing societies, limitations in instrumental activities of daily living (IADL) will become increasingly prevalent among community-dwelling older adults. Studies among European older adults showed that the prevalence of one or more IADL limitations increases from 17 to 54% among adults aged 65 years or older up to >90% among adults aged 90 years or older [1–3]. Such limitations are associated with a loss of independent living and high healthcare costs. Policy aimed at improving independent living of older persons coincides with the wish of older persons to live independently for as long as possible, in which the built environment may play an important role.

The physical design of older adults’ residential areas is suggested to contribute to independent living in several ways [4]. A safe and attractive residential area, and the nearby presence of shops and facilities, may increase independent living, as older adults are more likely to be able to do their daily groceries and to visit a hairdresser or pharmacy, independent of help from others. Current literature indeed shows that aesthetics (e.g. green spaces), destinations (e.g. grocery stores), and safety (e.g. lighting) are associated with less disabilities [5]. Previous studies exploring associations between residential area characteristics and disabilities have shown mixed results [6, 7]. These studies generally used cross-sectional designs which may weaken associations with residential area characteristics, since disabilities can fluctuate over time [8]. Including repeatedly measured disabilities in a relatively short period captures this fluctuation, and may therefore provide greater reliability of estimates resulting in more robust associations. Importantly, they should not by definition be interpreted as a “real” change.

Physical activity (PA) has shown to slow the progression of disability by decreasing functional limitations. As older persons spend more time being physically active outside than inside their homes [9], transport-related PA (TPA) may play an important role in the prevention of disabilities. A high ‘walkable’ residential area may promote walking for recreation and transport, which helps older adults to stay physically fit and live longer independently [6, 7]. Highly aesthetic residential areas and residential areas with many functional features (e.g. benches) or facilities are found to be associated with more minutes of transport-related walking [10]. Because older adults use residential areas for activities in daily life [11], transport-related physical activity (TPA) is thought to play an important role in the pathway between area characteristics and disabilities.

This study adds knowledge by investigating the association between residential area characteristics and repeatedly measured disabilities to better capture random fluctuation, and by investigating whether associations, if any, are mediated by TPA levels.

## Methods

### Design

Data from the Dutch ELANE study (2011–2013) were used. This longitudinal study aimed at studying associations between residential area characteristics and PA, independent living, and quality of life among adults aged 65 years and older living in Spijkenisse, a middle-sized town in the Rotterdam area. Community-dwelling older adults were randomly selected from the municipal register of Spijkenisse. Of the 430 persons interviewed face-to-face at baseline (T0), 277 (response 64.4%) were again interviewed by telephone 9 months later (T1). Some participants lacked data on residential area characteristics (n = 5) or disabilities at follow-up (n = 1), and therefore data of 271 persons were eligible for analyses. A more extensive description of the ELANE study can be found elsewhere [10].

### Disabilities

Disabilities were measured at baseline and follow-up by the Lawton and Brody scale [12], a reliable and moderately strong predictor of functioning [12–14]. Participants were asked whether they needed help with the following eight IADL activities: using the telephone, travelling (e.g. public transport), grocery shopping, preparing a meal, household tasks, taking medicines, finances, and doing laundry. All items had answering categories no (0) and yes (1), therefore sum scores could range between 0 and 8.

### Transport-related physical activity

Three repeatedly measured TPA-outcomes were included in the analyses: walking for transport, cycling for transport, and a combination of the two (further referred to as walking, cycling, and total TPA). These were based on questions from the Physical Activity Questionnaire in the LASA study (LAPAQ), a valid and reliable instrument to measure PA among older adults [15, 16]. We calculated total minutes of walking within the last 2 weeks by multiplying the answers to the following questions: ‘On how many days did you walk for transport in the past 2 weeks?’, and ‘How long did you walk for transport on average per day?’ Total minutes cycling were calculated based on similar questions for cycling. Total TPA was derived by summing minutes of walking and minutes of cycling. Because 18.1 and 42.6% of the study sample reported walking or cycling time of 0 min at baseline, and respectively 19.9 and 46.1% at follow-up, total walking time, total cycling time, and total TPA time were logtransformed. To meaningfully interpret the results, coefficients and CIs were retransformed after the statistical analyses.

### Residential area characteristics

Table 1 shows the ELANE street audit instrument which was used to collect data on residential area characteristics (carried out between June and October 2012) [10]. Sum scores were calculated for aesthetics, functional features, safety, and the presence of destinations by taking together separate items, as suggested by the framework of Pikora et al. [17].

**Table 1:** Street audit instrument to assess area characteristics, the ELANE study

Area characteristic	Score		
	0	1	2
Aesthetics (range 0–22)			
Litter	Much	Little	Absent
Dog waste	Much	Little	Absent
Graffiti	Much	Little	Absent
Park	Absent		Present
Maintenance benches	Insufficient/n.a.	Reasonable	Sufficient
Maintenance sidewalk(s)	Insufficient/n.a.	Reasonable	Sufficient
Maintenance street	Insufficient	Reasonable	Sufficient
Trees	None	Few	Many
Gardens	None	Few	Many
Other green	Absent	Partly	Mainly
Water	Absent	Partly	Mainly
Functional (range 0–14)			
Sidewalk side 1	Absent	<2 m	≥2 m
Sidewalk side 2	Absent	<2 m	≥2 m
Obstacles sidewalk(s)	Many/n.a.	Few	None
Flatness walking surface	Insufficient	Reasonable	Sufficient
Curb cuts	Insufficient/n.a.	Reasonable	Sufficient
Bench(es)	None	One	More than one
Wastebin(s)	None	One	More than one
Safety (range 0–16)			
Crossings	Absent	Without traffic light(s)	With traffic light(s)
Speed limiters	None	One	More than one
Lighting	Insufficient	Reasonable	Sufficient
Supervision	Insufficient	Reasonable	Sufficient
Ground-level houses	None	Few	Many
Upper-level houses	None	Few	Many
Bicycle lane(s)	Absent	Not seperated from carlane	Seperated from carlane
Traffic speed limit^a^	Walking path	15 km road	50 km road
Destinations (range 0–15)			
ATM	Absent	Present	
Letterbox	Absent	Present	
Bus stop^b^	Absent	More than one	
Supermarket	Absent	Present	
Bakery	Absent	Present	
Vegetable store	Absent	Present	
Butcher	Absent	Present	
Other shops	Absent	Present	
Shopping center	Absent	Present	
Hairdresser	Absent	Present	
Café	Absent	Present	
Nursing home	Absent	Present	
Pharmacy	Absent	Present	
Community center	Absent	Present	
Sport facility	Absent	Present	

^a^Combined walking/cycle path scored 0.5; a 30 km road scored 1.5

^b^One bus stop scored 0.5

Since the influence of residential area characteristics on health outcomes depends on the size of the area under study [18], we created road network buffers around each participant’s home including all routes from a participant’s home to streets up to 400, 800, and 1200 m. Road network buffers provide a more accurate exposure to environmental characteristic than traditional neighbourhood boundaries [19]. Scores for aesthetics, functional features, and safety of all audited streets within a buffer were summed and divided by the total number of streets audited in that buffer, resulting in average street scores for each buffer. For destinations, the number of destinations of all the streets in each buffer were summed [10]. For the analyses, longitudinal data were created assuming that the residential area characteristics remained stable over 9 months.

### Statistical analyses

Descriptive analyses included Chi square tests and t tests to explore sex and age differences between those included (i.e. those participating at both T0 and T1) and those excluded from the main analyses (i.e. lost to follow-up) in terms of demographics, disabilities, and TPA.

**Fig. 1:**
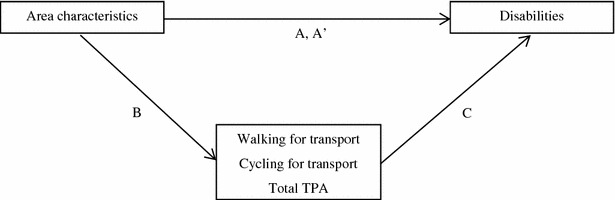
Conceptual model of the mediation analyses (based on Baron and Kenny, 1986)

Associations between residential area characteristics (aesthetics, functional features, safety, and destinations) and disabilities were tested, followed by analyses to investigate whether TPA mediated this association following conventional rules of mediation analysis as described by Baron and Kenny [20]. We subsequently tested the pathways A, B, C and A′ as shown in Fig. 1.

The proportion of persons reporting to have no disabilities at both T0 and T1 was 56.8%. An additional 9.6% of the participants reported no disabilities at T0 only, and another 6.3% reported no disabilities at T1 only. This suggests that many older adults did not experience any limitations in IADL. While some persons reporting no disabilities are “close” to having disabilities, others may still be far away from becoming functionally limited. As such, disabilities can be seen as an underlying latent variable with an unrestricted range, of which the observed outcome is a truncated version [21]. Tobit regression models are suitable for repeatedly measured data and take into account such censored data. Furthermore, longitudinal tobit regression models take into account correlated observations over time within persons. Therefore, multivariate longitudinal sex- and age adjusted tobit regression analyses were conducted to test associations between residential area characteristics and disabilities (pathway A). Associations between area characteristics and TPA (pathway B) were explored by using Generalized Estimating Equations [22] since it is unlikely that the TPA data was censored. Multivariate longitudinal sex–age and for area characteristics adjusted tobit regression analyses were conducted to test associations between TPA and disabilities (pathway C).

Educational level was excluded from analyses because no association was found with disability level.

The longitudinal tobit model can be formulated mathematically as follows [21]:

$$\begin{aligned} & {\text{y}}_{\text{ij}}^{*} |{\text{b}}_{\text{i}} = {\text{x}}_{\text{ij}}^{{\prime }}\upbeta + {\text{b}}_{\text{i}} + {\text{e}}_{\text{ij}} , \quad {\text{e}}_{\text{ij}} \sim\,{\text{N}}(0,\upsigma^{2} ) \\ & {\text{b}}_{\text{i}} \sim\,{\text{N(}}0,{\text{D)}} \\ \end{aligned}$$

in which y^*^ is a random latent variable that is not censored, β is the parameter, b_i_ is the case-specific random intercept with variance D, i refers to case i, j to the jth measurement within case i.

Finally, mediation of the association between area characteristics and disabilities by TPA was investigated (pathway A′). Analyses were performed by using STATA 14.1. Before the regression analyses were performed, panel data were defined (including 271 cases over 2 time periods, resulting in 271 × 2 observations). P values of 0.05 or lower were considered to be significant.

## Results

### Sample characteristics

Persons lost during follow-up were more often female, and reported on average more minutes walking than the study sample. No differences were found in the composition of both groups by age, minutes of cycling, and disabilities. At T0, 33.6% of the study sample had one or more disabilities. Although no difference was found between the mean number of disabilities at T0 and T1, after 9 months, 16.2% of the study sample had developed disabilities and 12.9% had recovered from disabilities. Also, total minutes of walking, cycling, and total TPA did not differ significantly between T0 and T1 (Table 2). Table 3 shows the scores for residential area characteristics per street for each buffer size. The average scores for aesthetics, functional features, and safety decreased slightly with increasing buffer size; the accumulated number of destinations within a buffer increased with increasing buffer size.

**Table 2:** Descriptive characteristics of the study sample at baseline and 9 months follow-up (N = 271)

		Total (N = 271)
Sex T0	Females	49.1%
Age T0	Mean	74.6 years
Disabilities T0 (range 0–8)	One or more	33.6%
	Mean number of disabilities	0.71 ± 1.35
Disabilities T1 (range 0–8)	One or more	36.9%
	Mean number of disabilities	0.73 ± 1.25
TPA T0 (minutes per 2 weeks)	Walking	344.5 ± 423.8
	Cycling	165.3 ± 248.3
	Total	509.8 ± 517.8
TPA T1 (minutes per 2 weeks)	Walking	349.4 ± 445.7
	Cycling	180.8 ± 357.0
	Total	530.2 ± 601.1

**Table 3:** Residential area characteristics of the four buffer zones

Area characteristics	Area		
	400 m	800 m	1200 m
Number of observed streets	39 ± 13	138 ± 40	294 ± 86
Aesthetics (range 0–22)	11.9 ± 0.9	11.8 ± 0.7	11.7 ± 0.6
Functional features (range 0–14)	5.8 ± 1.7	5.4 ± 1.1	5.3 ± 0.9
Safety (range 0–16)	6.1 ± 1.0	6.0 ± 0.7	5.9 ± 0.6
Destinations (range 0–∞)	10 ± 9	30 ± 16	57 ± 22

### Area characteristics and disabilities

We subsequently tested the pathways A, B, C and A′ (Fig. 1). Within all buffers, area aesthetics showed comparable associations with disabilities, but was only significant in the 400 m buffer in which an increase in the aesthetics score of one point was associated with 0.86 less disabilities (95% CI −1.47 to −0.26; p < 0.05; pathway A) (Table 4). No associations for other area characteristics within the 400 m buffer, or for area characteristics of the 800 and 1200 m buffers with disabilities were found, although the association between aesthetics and disabilities in the 800 m was close to significant.

**Table 4:** Age and sex adjusted associations between area characteristics and disabilities (pathway A and A′; N = 271)

Area	Area characteristic^a^	Pathway A			Pathway A′								
		Disabilities			Disabilities adjusted for transport-related walking			Disabilities adjusted for transport-related cycling			Disabilities adjusted for total TPA		
		β	(95% CI)	p	β	(95% CI)	p	β	(95% CI)	p	β	(95% CI)	p
400 m	Aesthetics	−0.86*	(−1.47 to −0.26)	0.01	−0.77*	(−1.34 to −0.19)	0.01	−0.71*	(−1.26 to −0.16)	0.01	−0.69*	(−1.21 to −0.16)	0.01
	Functional features	0.27	(−0.09 to 0.64)	0.14	0.22	(−0.13 to 0.57)	0.22	0.32	(−0.01 to 0.65)	0.06	0.22	(−0.10 to 0.53)	0.17
	Safety	0.22	(−0.35 to 0.78)	0.45	0.22	(−0.32 to 0.76)	0.43	0.06	(−0.46 to 0.57)	0.84	0.17	(−0.32 to 0.66)	0.49
	Destinations	−0.03	(−0.08 to 0.02)	0.21	−0.02	(−0.07 to 0.02)	0.28	−0.03	(−0.07 to 0.01)	0.15	−0.02	(−0.06 to 0.02)	0.26
800 m	Aesthetics	−0.97	(−1.96 to 0.02)	0.05	−0.81	(−1.75 to 0.13)	0.09	−0.83	(−1.72 to 0.07)	0.07	−0.66	(−1.51 to 0.19)	0.13
	Functional features	0.35	(−0.30 to 0.99)	0.29	0.32	(−0.30 to 0.93)	0.31	0.40	(−0.18 to 0.99)	0.18	0.28	(−0.27 to 0.84)	0.31
	Safety	0.04	(−0.78 to 0.86)	0.93	−0.06	(−0.84 to 0.71)	0.88	−0.07	(−0.81 to 0.67)	0.86	−0.13	(−0.84 to 0.57)	0.71
	Destinations	−0.00	(−0.03 to 0.02)	0.74	−0.00	(−0.03 to 0.02)	0.93	−0.01	(−0.03 to 0.02)	0.51	0.00	(−0.02 to 0.02)	0.99
1200 m	Aesthetics	−1.21	(−2.74 to 0.32)	0.12	−0.85	(−2.31 to 0.62)	0.26	−1.18	(−2.66 to 0.30)	0.12	−0.48	(−1.81 to 0.85)	0.48
	Functional features	0.62	(−0.64 to 1.88)	0.34	0.48	(−0.72 to 1.68)	0.43	0.63	(−0.60 to 1.85)	0.32	0.26	(−0.83 to 1.35)	0.64
	Safety	0.01	(−1.05 to 1.08)	0.98	−0.13	(−1.15 to 0.88)	0.80	−0.01	(−1.04 to 1.02)	0.99	−0.21	(−1.13 to 0.71)	0.65
	Destinations	−0.01	(−0.03 to 0.01)	0.50	−0.01	(−0.02 to 0.01)	0.57	−0.01	(−0.03 to 0.01)	0.44	−0.00	(−0.02 to 0.01)	0.70

* p < 0.05

^a^Adjustments were made for age, sex, and the other area characteristics

### Area characteristics and TPA

For all three buffer sizes, associations between area characteristics with minutes walking and cycling were found (pathway B). In the 400 and 1200 m buffers, higher safety scores were associated with less cycling and walking respectively. With increasing buffer size, the strength of the association between aesthetics and minutes walking increased which was found significant in the two largest buffers. Only in the 1200 m buffer, a significant association was found with total TPA: higher scores on aesthetics were associated with more total TPA (Table 5).

**Table 5:** Age and sex adjusted associations between area characteristics and TPA (pathway B; N = 271)

Area	Area characteristic^a^	TPA								
		Walking			Cycling			Total TPA		
		β	(95% CI)	p	β	(95% CI)	p	β	(95% CI)	p
400 m	Aesthetics	1.34	(0.86–2.11)	0.19	1.44	(0.85–2.46)	0.17	1.35	(0.91–2.00)	0.13
	Functional features	0.77	(0.60–1.00)	0.05	1.27	(0.93–1.72)	0.13	0.92	(0.73–1.15)	0.44
	Safety	1.09	(0.72–1.65)	0.68	0.56*	(0.34–0.91)	0.02	0.91	(0.63–1.30)	0.59
	Destinations	1.02	(0.98–1.05)	0.32	0.98	(0.95–1.03)	0.45	1.01	(0.98–1.04)	0.59
800 m	Aesthetics	2.06*	(1.00–4.26)	0.05	1.10	(0.46–2.62)	0.82	1.77	(0.94–3.35)	0.08
	Functional features	0.85	(0.54–1.34)	0.47	1.42	(0.82–2.46)	0.21	0.93	(0.62–1.39)	0.72
	Safety	0.58	(0.32–1.05)	0.07	0.70	(0.34–1.42)	0.32	0.62	(0.37–1.05)	0.07
	Destinations	1.02	(1.00–1.04)	0.07	0.98	(0.96–1.00)	0.12	1.01	(0.99–1.03)	0.23
1200 m	Aesthetics	4.53*	(1.49–13.79)	0.01	1.53	(0.53–4.44)	0.43	4.26*	(1.61–11.30)	0.00
	Functional features	0.60	(0.24–1.47)	0.26	0.81	(0.34–1.92)	0.63	0.51	(0.23–1.12)	0.09
	Safety	0.45*	(0.21–0.98)	0.04	0.77	(0.36–1.62)	0.49	0.54	(0.28–1.08)	0.08
	Destinations	1.01	(0.99–1.02)	0.42	1.00	(0.98–1.01)	0.56	1.01	(0.99–1.02)	0.30

Beta coefficients less than 1 represent negative associations, beta coefficients more than 1 represent positive associations

* p < 0.05

^a^Adjustments were made for age, sex, and the other area characteristics

### TPA and disabilities

Both higher levels of walking and cycling were associated with less disabilities (pathway C; Table 6). An increase of 10 min walking per 2 weeks was associated with 0.01 less disabilities (p < 0.001). An increase of 10 min cycling was associated with 0.02 less disabilities (p < 0.001). An increase of 10 min total TPA was associated with 0.01 less disabilities (p < 0.001).

**Table 6:** Associations between TPA and disabilities adjusted for area characteristics (pathway C; N = 271)

	Disabilities		
	β	(95% CI)	p
Adjusted for area characteristics within 400 m			
Walking	−0.01*	(−0.02 to −0.01)	0.00
Cycling	−0.02*	(−0.03 to −0.01)	0.00
Total TPA	−0.01*	(−0.02 to −0.01)	0.00
Adjusted for area characteristics within 800 m			
Walking	−0.01*	(−0.02 to −0.01)	0.00
Cycling	−0.02*	(−0.03 to −0.01)	0.00
Total TPA	−0.01*	(−0.02 to −0.01)	0.00
Adjusted for area characteristics within 1200 m			
Walking	−0.01*	(−0.02 to −0.01)	0.00
Cycling	−0.02*	(−0.03 to −0.01)	0.00
Total TPA	−0.01*	(−0.02 to −0.01)	0.00

* p < 0.05

### Mediation

Inclusion of minutes walking and cycling separately to the model in which aesthetics of the 400 m buffer was related to disabilities, resulted in minor attenuations of the coefficient (pathway A′; Table 4). Adding total minutes TPA resulted in the largest attenuation: the regression coefficient changed from −0.86 to −0.69 (95% CI −1.21 to −0.16, p < 0.05). Except for the coefficients for safety in the 800 and 1200 m buffer, all coefficients representing associations between area characteristics and disabilities became closer to zero once TPA outcomes were added to the models.

## Discussion

Of the four area characteristics under study, only higher scores on area aesthetics within a 400 m buffer were associated with less disabilities. While transport-related walking and cycling were associated with residential area characteristics and disabilities, only a small part of the association between aesthetics and disabilities was mediated by these factors.

Older adults living in areas with good aesthetics reported less disabilities, which is supported by other studies showing that those residing in areas with more green spaces and better neighbourhood maintenance (e.g. maintenance of streets and pavements) had lower levels of disabilities [5, 23]. We did not find associations with disabilities for the other area characteristics, which is in contrast to literature showing that more functional features (e.g. presence of sidewalks), traffic-related safety, and destinations (e.g. grocery stores) are associated with lower levels of disabilities [5, 24]. Differences in results may be due to different measures of disabilities and area characteristics, but may also reflect that the influence of the built environment on disabilities varies by country. In a sensitivity analysis, area characteristics were linked to the specific IADL-items regarding ‘limitations in travelling (e.g. by public transport)’ and ‘limitations in grocery shopping’ which are perhaps more directly related to mobility as compared to some elements of our IADL scale. Associations with area characteristics were only found for travelling: higher scores on aesthetics within all buffers were associated with less limitations in travelling (beta coefficient up to −0.26 in the 1200 m buffer, CI −0.42 to −0.11; p < 0.05). This beta coefficient showed the highest drop (to −0.20) after total TPA was added to the model (“Appendix”). Based on a systematic review it has been recommended to revise built environment instrument including more disability-specific items [25]. Although the measure for functional features the ELANE neighbourhood scan did include width of side-walks and the presence of curb cuts, the scan for example did not include availability of signage or accessibility of green spaces or facilities [25]. Previous work based on ELANE baseline data showed a positive association between the presence of destinations and walking for transport [10]. We did not find this association in our current study, which may be caused by a lack of power due to the smaller study population.

A negative association was found between safety and transport-related walking in the 1200 m buffer. There is inconsistent evidence for associations between safety and walking which could be attributed to the complexity of measuring safety [26]. In a sensitivity-analysis we split our safety measure into a set of traffic safety items (i.e. presence of crossings, speed limiters, bicycle lanes, and traffic speed limits) and a set of social safety items (i.e. presence of lighting, supervision, houses, and apartments). Within the 400 m buffer, no significant associations were found between both safety measures and cycling (in contrast to the main finding presented in Table 5). Within the 1200 m buffer, higher scores for traffic safety were associated with less cycling. To improve research on safety and PA, Foster and Giles-Corti [26] suggested to combine objective measurement of safety with subjective measures of safety in which besides judgements (e.g. crime is a problem in the neighbourhood), and emotional responses (e.g. being fearful about the crime) should also be taken into account [26].

Although most associations were found non-significant, the results of the mediation analyses indicated the possible role of TPA in the associations between area characteristics and disabilities. TPA only partly explained the association between aesthetics and disabilities which may be due to the small effect size of the association between TPA and disabilities. The finding that an increase of 10 min cycling per 2 weeks was associated with 0.02 less disabilities, implicates that for example an increase of 25 min cycling per week may decrease disabilities (range 0–8) with 0.1. Other studies did also find effects of increasing minutes of physical activity per week. For example, Rist et al. found physical inactivity to be associated with 0.14 more IADL limitations over 2 years [27]. Another study by Boyle et al. showed that among non-disabled persons, the risk to develop IADL disability decreased with 7% for each additional hour of physical activity per week [28]. Despite the mixed findings of studies on the association between PA and disability, as some do not find significant associations, our findings relate to the thought that physical activity is modestly associated with disability [28]. TPA only partly explained the association between aesthetics and disabilities. It is of interest to investigate other possible mediating factors such as other health behaviors (e.g. recreational PA, nutrition), mental health, and social participation, which may be promoted by area characteristics [29, 30] and could potentially prevent disabilities [31, 32].

This study is among the first to study the role of area characteristics for disability among older persons and the role of transport-related physical activity. A main strength of the study was the use of repeatedly measured disabilities which was justified by the finding of substantial variation in disabilities between baseline and follow-up. For this purpose we applied longitudinal logit regression models which are able to capture these random fluctuations. The variation could be due to real differences in disabilities at both moments in time; previous studies also showed that the development of disabilities is a dynamic process [8]. The variation could also result from random measurement error of disabilities. Such measurement error increases the likelihood of bias towards the null in studies using disabilities measured at a single time. Although it is possible to recover from disabilities, older adults who have recovered are at high risk of recurrent disabilities [33].

Several limitations should also be mentioned. Firstly, 153 participants (35.6%) were lost to follow-up because they were not willing to participate (n = 135), unreachable by telephone (n = 11), had health problems (n = 3) or provided other reasons (n = 4). As compared to the overall sample at baseline, those lost to follow up were more often women, and reported more minutes walking at baseline, but did not differ in disability scores. It may limit the generalizability of the study results as those being most physically active may have been underrepresented in the study sample. The effect on the main outcome, pathway A, is expected to be limited as no differences were found in disability scores. Secondly, study participants were interviewed face-to-face at baseline and by telephone at follow-up. Although we cannot exclude the possibility that different methods may have resulted in over- or underestimations, the overall impact may be limited since the same procedure was used for all participants, i.e. both interviews asked for self-reported levels of PA and disabilities. Thirdly, the association between area characteristics and cycling for transport may be underestimated since 23.8% of the data used to measure area characteristics was related to walking only (i.e. characteristics of walking paths). Moreover, it is suggested to use larger longitudinal datasets and to use more accurate measurement of area characteristics related to cycling, in order to get more insight in associations between the built environment and disabilities and the role of TPA.

Fourthly, it should be recognized that causality cannot be proven, since findings presented are based on an observational study. Self-selection may have played a role in the interpretation of associations as active older adults self-selecting themselves into areas conducive for PA. Additional analyses showed that self-selection probably did not affect the results, as only 6.3% (n = 17) had moved to their current residence in the past 5 years. The most prevalent reason for moving was a lower level of maintenance of the house (n = 9). One person reported a reason related to the built environment, i.e. because of a more attractive neighbourhood. Associations between TPA and disability may be confounded by other lifestyle factors such as smoking and BMI [34], and health-related factors such as mental health, as for example depressive persons are more likely to be less physically active and to develop disabilities as compared to non-depressed persons [35, 36]. Finally, to capture the development of disabilities more accurately, it is suggested to study disabilities over a longer time-period.

### Conclusions

Better aesthetic features of the area close by the residences of community-dwelling older adults were associated with less disabilities, but only a small part of this association seemed to be mediated by TPA. Higher scores for aesthetics and safety were associated with higher levels of TPA, and TPA was associated with disabilities. Preventive measures to reduce or prevent disabilities may include area characteristic improvements, however more research is needed to strengthen our results.

## Appendix: Results of pathway A, A′ and C for IADL items grocery shopping and travelling

See Tables 7, 8, 9, and 10.

**Table 7:** Age and sex adjusted associations between area characteristics and grocery shopping (pathway A and A′; N = 271)

Area	Area characteristic^a^	Pathway A			Pathway A′								
		Grocery shopping			Grocery shopping adjusted for transport-related walking			Grocery shopping adjusted for transport-related cycling			Grocery shopping adjusted for total TPA		
		β	(95% CI)	p	β	(95% CI)	p	β	(95% CI)	p	β	(95% CI)	p
400 m	Aesthetics	−0.05	(−0.10 to 0.01)	0.08	−0.04	(−0.10 to 0.01)	0.12	−0.04	(−0.09 to 0.01)	0.12	−0.04	(−0.09 to 0.01)	0.15
	Functional features	−0.00	(−0.03 to 0.03)	0.88	−0.01	(−0.04 to 0.02)	0.64	0.00	(−0.03 to 0.03)	0.92	−0.01	(−0.03 to 0.02)	0.72
	Safety	0.03	(−0.02 to 0.08)	0.31	0.03	(−0.02 to 0.08)	0.27	0.02	(−0.03 to 0.06)	0.51	0.02	(−0.03 to 0.07)	0.36
	Destinations	−0.00	(−0.01 to 0.00)	0.55	−0.00	(−0.00 to 0.00)	0.66	−0.00	(−0.01 to 0.00)	0.46	−0.00	(−0.00 to 0.00)	0.63
800 m	Aesthetics	−0.01	(−0.10 to 0.07)	0.75	0.00	(−0.09 to 0.09)	0.99	−0.01	(−0.10 to 0.07)	0.78	0.00	(−0.08 to 0.09)	0.88
	Functional features	−0.02	(−0.08 to 0.03)	0.46	−0.02	(−0.08 to 0.03)	0.39	−0.01	(−0.07 to 0.04)	0.59	−0.02	(−0.08 to 0.03)	0.39
	Safety	0.02	(−0.06 to 0.09)	0.66	0.00	(−0.07 to 0.08)	0.88	0.01	(−0.06 to 0.08)	0.78	−0.00	(−0.07 to 0.07)	0.98
	Destinations	0.00	(−0.00 to 0.00)	0.82	0.00	(−0.00 to 0.00)	0.60	−0.00	(−0.00 to 0.00)	0.97	0.00	(−0.00 to 0.00)	0.58
1200 m	Aesthetics	−0.00	(−0.14 to 0.13)	0.97	0.03	(−0.11 to 0.16)	0.69	0.00	(−0.13 to 0.14)	0.99	0.05	(−0.08 to 0.18)	0.45
	Functional features	−0.03	(−0.14 to 0.08)	0.56	−0.04	(−0.15 to 0.07)	0.44	−0.03	(−0.14 to 0.08)	0.55	−0.06	(−0.16 to 0.05)	0.29
	Safety	0.01	(−0.08 to 0.11)	0.79	−0.00	(−0.10 to 0.09)	0.95	0.01	(−0.08 to 0.11)	0.82	−0.01	(−0.10 to 0.08)	0.84
	Destinations	0.00	(−0.00 to 0.00)	0.18	0.00	(−0.00 to 0.00)	0.13	0.00	(−0.00 to 0.00)	0.19	0.00	(−0.00 to 0.00)	0.09

* p < 0.05

^a^Adjustments were made for age, sex, and the other area characteristics

**Table 8:** Associations between TPA and grocery shopping adjusted for area characteristics (pathway C; N = 271)

	Grocery shopping		
	β	(95% CI)	p
Adjusted for area characteristics within 400 m			
Walking	−0.02*	(−0.03 to −0.01)	0.00
Cycling	−0.02*	(−0.03 to −0.01)	0.00
Total TPA	−0.04*	(−0.05 to −0.02)	0.00
Adjusted for area characteristics within 800 m			
Walking	−0.02*	(−0.03 to −0.01)	0.00
Cycling	−0.02*	(−0.03 to −0.01)	0.00
Total TPA	−0.04*	(−0.05 to −0.02)	0.00
Adjusted for area characteristics within 1200 m			
Walking	−0.02*	(−0.03 to −0.01)	0.00
Cycling	−0.01*	(−0.01 to 0.00)	0.00
Total TPA	−0.04*	(−0.05 to −0.02)	0.00

* p < 0.05

**Table 9:** Age and sex adjusted associations between area characteristics and travelling (pathway A and A′; N = 271)

Area	Area characteristic^a^	Pathway A			Pathway A′								
		Travelling			Travelling adjusted for transport-related walking			Travelling adjusted for transport-related cycling			Travelling adjusted for total TPA		
		β	(95% CI)	p	β	(95% CI)	p	β	(95% CI)	p	Β	(95% CI)	p
400 m	Aesthetics	−0.11*	(−0.17 to −0.05)	0.00	−0.11*	(−0.17 to −0.05)	0.00	−0.10*	(−0.16 to −0.04)	0.00	−0.10*	(−0.16 to −0.04)	0.00
	Functional features	0.03	(−0.00 to 0.07)	0.07	0.03	(−0.01 to 0.06)	0.10	0.04*	(0.01 to 0.07)	0.02	0.03	(−0.00 to 0.06)	0.08
	Safety	0.03	(−0.02 to 0.09)	0.20	0.04	(−0.02 to 0.09)	0.17	0.02	(−0.04 to 0.07)	0.50	0.03	(−0.02 to 0.09)	0.21
	Destinations	−0.01*	(−0.01 to −0.00)	0.03	−0.01*	(−0.01 to −0.00)	0.04	−0.01*	(−0.01 to −0.00)	0.01	−0.00*	(−0.01 to −0.00)	0.03
800 m	Aesthetics	−0.17*	(−0.27 to −0.07)	0.00	−0.16*	(−0.26 to −0.06)	0.00	−0.17*	(−0.26 to −0.08)	0.00	−0.15*	(−0.24 to −0.06)	0.00
	Functional features	0.03	(−0.03 to 0.09)	0.37	0.03	(−0.04 to 0.09)	0.40	0.04	(−0.02 to 0.10)	0.18	0.03	(−0.03 to 0.08)	0.38
	Safety	0.10*	(0.02 to 0.18)	0.02	0.09*	(0.01 to 0.17)	0.02	0.09*	(0.01 to 0.17)	0.02	0.08*	(0.01 to 0.16)	0.04
	Destinations	−0.00	(−0.00 to 0.00)	0.31	−0.00	(−0.00 to 0.00)	0.41	−0.00	(−0.00 to 0.00)	0.12	−0.00	(−0.00 to 0.00)	0.45
1200 m	Aesthetics	−0.26*	(−0.42 to −0.11)	0.00	−0.24*	(−0.40 to −0.09)	0.00	−0.26*	(−0.41 to −0.10)	0.00	−0.20*	(−0.34 to −0.06)	0.01
	Functional features	0.09	(−0.04 to 0.22)	0.16	0.08	(−0.04 to 0.21)	0.19	0.09	(−0.04 to 0.21)	0.17	0.06	(−0.05 to 0.18)	0.30
	Safety	0.13*	(0.02 to 0.23)	0.02	0.11*	(0.01 to 0.22)	0.04	0.12*	(0.02 to 0.23)	0.03	0.10*	(0.00 to 0.20)	0.04
	Destinations	−0.00	(−0.00 to 0.00)	0.07	−0.00	(−0.00 to 0.00)	0.08	−0.00	(−0.00 to 0.00)	0.06	−0.00	(−0.00 to 0.00)	0.10

* p < 0.05

^a^Adjustments were made for age, sex, and the other area characteristics

**Table 10:** Associations between TPA and travelling adjusted for area characteristics (pathway C; N = 271)

	Travelling		
	β	(95% CI)	P
Adjusted for area characteristics within 400 m			
Walking	−0.02*	(−0.03 to −0.00)	0.01
Cycling	−0.03*	(−0.04 to −0.02)	0.00
Total TPA	−0.04*	(−0.06 to −0.03)	0.00
Adjusted for area characteristics within 800 m			
Walking	−0.01*	(−0.03 to −0.00)	0.01
Cycling	−0.03*	(−0.04 to −0.02)	0.00
Total TPA	−0.04*	(−0.06 to −0.03)	0.00
Adjusted for area characteristics within 1200 m			
Walking	−0.01*	(−0.03 to −0.00)	0.02
Cycling	−0.02*	(−0.02 to −0.01)	0.00
Total TPA	−0.04*	(−0.06 to −0.03)	0.00

* p < 0.05
